# Optimizing Hybrid *de Novo* Transcriptome Assembly and Extending Genomic Resources for Giant Freshwater Prawns (*Macrobrachium rosenbergii*): The Identification of Genes and Markers Associated with Reproduction

**DOI:** 10.3390/ijms17050690

**Published:** 2016-05-07

**Authors:** Hyungtaek Jung, Byung-Ha Yoon, Woo-Jin Kim, Dong-Wook Kim, David A. Hurwood, Russell E. Lyons, Krishna R. Salin, Heui-Soo Kim, Ilseon Baek, Vincent Chand, Peter B. Mather

**Affiliations:** 1 Centre for Tropical Crops and Biocommodities, Science and Engineering Faculty, Queensland University of Technology, Queensland 4000, Australia; 2 Korean Bioinformation Center, Korea Research Institute of Bioscience and Biotechnology, Daejeon 305806, Korea; cogate@kribb.re.kr; 3 Department of Bioinformatics, University of Science and Technology, Daejeon 305333, Korea; 4 Biotechnology Research Division, National Institute of Fisheries Science, Busan 46083, Korea; wj2464@korea.kr; 5 All Bio Technology Co., LTD, Internet Business Incubation Center, Mokweon University, Daejeon 302729, Korea; kdw9017@naver.com; 6 Earth, Environmental and Biological Sciences, Science and Engineering Faculty, Queensland University of Technology, Queensland 4000, Australia; d.hurwood@qut.edu.au (D.A.H.); v.chand@qut.edu.au (V.C.); p.mather@qut.edu.au (P.B.M.); 7 School of Veterinary Science, University of Queensland, Queensland 4067, Australia; r.lyons2@uq.edu.au; 8 School of Environment, Resources and Development, Asian Institute of Technology, Pathumthani 12120, Thailand; salinkr@gmail.com; 9 Department of Biological Sciences, College of Natural Sciences, Pusan National University, Busan 609735, Korea; khs307@pusan.ac.kr; 10 Division of Marine Technology, Chonnam National University, Yeosu 550250, Korea; chitago@naver.com

**Keywords:** *Macrobrachium rosenbergii*, crustacean, prawn, *de novo* assembly, hybrid transcriptome, optimization, reproduction

## Abstract

The giant freshwater prawn, *Macrobrachium rosenbergii*, a sexually dimorphic decapod crustacean is currently the world’s most economically important cultured freshwater crustacean species. Despite its economic importance, there is currently a lack of genomic resources available for this species, and this has limited exploration of the molecular mechanisms that control the *M. rosenbergii* sex-differentiation system more widely in freshwater prawns. Here, we present the first hybrid transcriptome from *M. rosenbergii* applying RNA-Seq technologies directed at identifying genes that have potential functional roles in reproductive-related traits. A total of 13,733,210 combined raw reads (1720 Mbp) were obtained from Ion-Torrent PGM and 454 FLX. Bioinformatic analyses based on three state-of-the-art assemblers, the CLC Genomic Workbench, Trans-ABySS, and Trinity, that use single and multiple *k*-mer methods respectively, were used to analyse the data. The influence of multiple *k*-mers on assembly performance was assessed to gain insight into transcriptome assembly from short reads. After optimisation, *de novo* assembly resulted in 44,407 contigs with a mean length of 437 bp, and the assembled transcripts were further functionally annotated to detect single nucleotide polymorphisms and simple sequence repeat motifs. Gene expression analysis was also used to compare expression patterns from ovary and testis tissue libraries to identify genes with potential roles in reproduction and sex differentiation. The large transcript set assembled here represents the most comprehensive set of transcriptomic resources ever developed for reproduction traits in *M. rosenbergii*, and the large number of genetic markers predicted should constitute an invaluable resource for future genetic research studies on *M*. *rosenbergii* and can be applied more widely on other freshwater prawn species in the genus *Macrobrachium*.

## 1. Introduction

For crustaceans, unlike most model organisms, genomic studies have lagged, and, currently, only limited genomic information exists for any target species. Despite this fact, some recent studies have made significant progress towards understanding the basic biology and genome make-up of certain crustacean species following high-throughput sequence analysis [1,2,3,4,5]. In particular, reproductive traits are considered to be very important production traits in the crustacean culture industry, so recent studies have been directed at understanding reproductive development in shrimp [2,3,4] that can allow reproduction to be manipulated by animal breeders in order to increase seed quality and to permit genetically improved lines to be developed. As a consequence, the focus of crustacean research that focuses on reproductive and developmental biology has changed from histological analyses to molecular studies [5,6,7,8].

The giant freshwater prawn *M*. *rosenbergii* has been domesticated for approximately 40 years and now supports one of the largest culture industries (global production of 234,206 tons) for any crustacean species farmed in inland waters across the world [9]. For *M. rosenbergii*, a number of recent studies have been conducted to study complex traits involved with metamorphosis, growth and muscle development, and effects of environmental stress on gene expression patterns including a neuropeptidome study [10,11,12,13]. Interest in the genetics of reproductive development and sex differentiation in this species, in particular the sexual dimorphism evident in males for growth rate, is motivated by potential practical and commercial applications, including the goal of producing monosex male progeny because males can grow to much larger individual size than females, and by fundamental scientific curiosity [14,15]. This viewpoint has been challenged, however, by Malecha *et al.* [16], who suggested that all-female culture could be more promising than all-male culture because of the evenness of female cohorts. In addition, while a recent study has indicated that individual reproductive quality is highly associated with individual growth performance in *M*. *rosenbergii* [17], the genetic factors that affect this relationship remains unknown. An initial step towards understanding the molecular basis of the genetic regulation of maturation has already been initiated for *M*. *rosenbergii* [18,19,20], and this has included a focus on the progress of sexual differentiation (*i.e.*, sex-linked markers) [21,22,23]. While understanding the molecular mechanisms of testicular and ovarian development in freshwater prawns is a crucial step to controlling gonadal development and growth, the majority of earlier molecular genomic studies of *M*. *rosenbergii* have been undertaken at a small scale and have only investigated a few putative genes potentially involved with reproductive development [20,22]. This contrasts with studies of other commercially important crab, prawn, and shrimp species [5,7,8]. Developing more sophisticated controls over sexual maturation, sex differentiation, and reproduction can potentially assist industry development and improve relative productivity of culture lines. Consequently, there is a need to identify and to characterise reproduction-related genes in *M*. *rosenbergii*, and to understand the molecular mechanisms of gonadal maturation, as this knowledge can help improve breeding practices and allow genetically improved strains to be developed more rapidly for the aquaculture industry.

Since a comprehensive genome sequence is not currently available for the target species, a next-generation sequencing (NGS) approach, specifically RNA-Seq applications, can be used for an efficient identification of molecular markers and transcripts involved in important biological processes. However, production of very large numbers of short reads generated with RNA-Seq can often make transcriptome assembly difficult due to issues associated with repeat sequencing, the potential for alternatively spliced transcripts, and a high variation in sequence coverage [24,25]. Thus, *de novo* assembly of a transcriptome is a commonly overlooked yet critical step, in particular when working with NGS data in species where a reference genome is not currently available. If NGS data are assembled accurately and efficiently, transcriptome analysis is advantageous and can provide the essential resources that allow functional elements of the genome to be characterised and mechanisms resolved [26]. While, to date, the ecological genetics research community has largely focused its attention on read length, assembly annotation, and single nucleotide polymorphism (SNP) identification, we believe that optimisation of data assembly often requires greater attention. Here, we address these needs by evaluating the performance of short read assemblers (CLC Genomic Workbench, Trans-ABySS and Trinity) in *de novo* transcriptome assembly of hybrid sequences (from combined 454 FLX and Ion-Torrent sequence data) where a reference genome was not available for the target species.

In the current study, we report deep sequencing of *M. rosenbergii* transcriptomes produced using two NGS platforms from RNA-Seq data (454 FLX and Ion-Torrent) from muscle, ovary, and testis tissue libraries as an initial step to obtain a large and reliable genomic/transcriptomic dataset. The goals of this study were (1) to develop an optimised *de novo* assembly by comparing and evaluating the usefulness of three different assemblers; (2) to identify putative genes with functional roles in *M*. *rosenbergii* reproductive development that can provide useful markers for understanding key processes; and (3) to discover a variety of markers potentially useful for genetic studies including simple sequence repeats (SSRs) and SNPs. Furthermore, we believe that identified key genes and mutations here can provide fundamental and significant information about reproductive development in the target species, *M*. *rosenbergii*.

## 2. Results

### 2.1. Transcriptome Sequencing

To obtain the most comprehensive transcriptome, an earlier 454 FLX [10] dataset was extended by adding the Ion-Torrent sequencing dataset from muscle, ovary, and testis tissues from the same 18 *M. rosenbergii* animals. In the case of Ion-Torrent, a total of four 316 chips were run which resulted in a total of 1475 Mbp averaging 113 bp in read length (Table 1). Ion-Torrent sequencing (average 368 Mbp per chip) generated more data with less time and labour costs than 454 FLX (average 250 Mbp per half plate). In terms of average read length, however, 454 FLX generated longer average read lengths than was evident with Ion-Torrent.

Combining the runs from both platforms produced a total of 1720 Mbp comprising 13,733,210 raw reads. More detailed explanation of the 454 FLX run can be found in our previous work [10] and Table 1. After the removal of all ambiguous nucleotides, low-quality reads (quality scores <20), and sequences less than 50 bp (454 FLX) and 30 bp (Ion-Torrent), over 0.75 million (454 FLX) and 11.14 million (Ion-Torrent) reads were obtained for further downstream analysis (Table 1). Total average GC content was 42.61% for raw reads and 43.30% for trimmed reads from combined platform runs. While different GC contents were observed due to two different sequencing technologies, ovary tissue showed significantly lower GC percentage compared with other sampled tissues. However, a cautious interpretation should be made due to a potential sequencing error rate from two different sequencing technologies. A more detailed explanation of each library is presented in Table 1. All raw reads (454 FLX and Ion-Torrent) were deposited in the NCBI and can be accessed in the Short Read Archive (SRA) under accession numbers SRX1077238.

### 2.2. Transcriptome de Novo Assembly and Comparison of Assemblers

**Figure 1:**
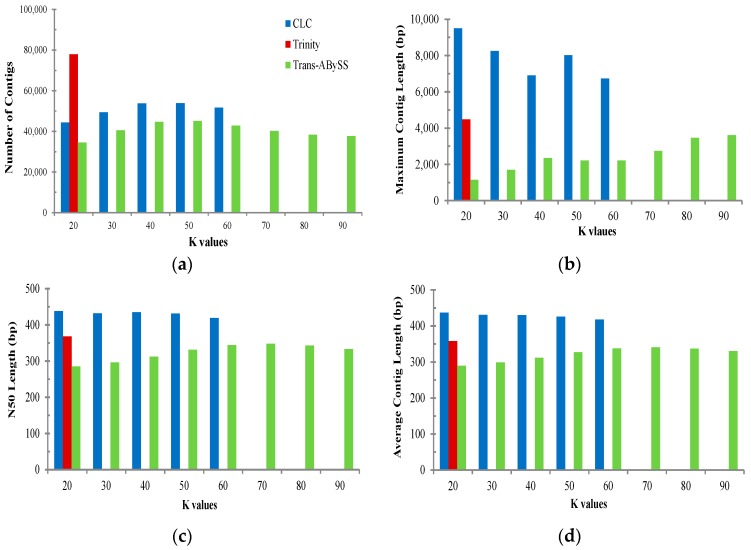
Comparison of *de novo* assemblies applying multi *K*-values. *K*-values in Trinity indicate 25-mer default value. (**a**) Number of cintigs; (**b**) Maximum contig length (bp); (**c**) N50 length (bp), and (**d**) Average contig length (bp).

The dataset was analysed applying three different assemblers, which include: CLC Genomics Workbench (v6.5), Trans-ABySS (v1.4.4), and Trinity (r2013-02-16) for *de novo* assembly. While the same de Bruijn graph algorithm was employed with all three assemblers, each treats sequencing errors, resolves ambiguities, and utilises read pair information differently [27], which results in different assembly outcomes. Since the multiple *k*-mer approach applied by the three assemblers has the capacity to produce a better assembly for transcripts from both high and low expressed genes, we applied different *k*-mer lengths with all three assemblers to choose the best *k*-mer length for *de novo* assembly. We first generated a dataset from the merged datasets from all 454 FLX and Ion-Torrent sequencing data. The outcomes with distinct *k*-mer values were compared using the four criteria, total contig number, N50 length, average contig length, and maximum contig length. It is generally acknowledged that larger values of these criteria potentially imply better assembly performance (but not for total contig number) [10,25]. The result showed that the values varied extensively according to the *k*-mer size in each assembly (Figure 1); thus, data from contigs longer than 200 bp were selected to evaluate assembly quality because results of the pre-run of the three assemblers consistently revealed that data including short contigs (<200 bp) yielded less accurate results than did data with longer contigs.

In order to evaluate the best assembler, four statistical criteria were compared for determining the relative quality of *de novo* transcriptome data. Of the three assemblers’ trialed, the 20-mer in the CLC assembly (using the optimised default parameter) produced the best *de novo* assembly of the three assemblers and resulted in 44,407 contigs with an average contig length of 437 bp and an N50 length of 438 bp. The Trinity assembly produced the next best result with a *k*-mer length of 25 (an average contig length of 358 bp and an N50 length of 368 bp) followed by the Trans-ABySS assembly with an optimum *k*-mer length of 75 (an average contig length of 341 bp and an N50 length of 348 bp). For the Trinity assembly, since only the default parameters were available (a *k*-mer length of 25), further comparison of *k*-mer performance was not applicable. While the total number of contigs generated with Trinity was higher than that with the other two assemblers, other criteria values were lower than that with CLC but higher than that with Trans-ABySS. For each library, *de novo* assembly with optimal setting parameters (*k*-mers) for the three assemblers produced similar overall assembly performance of the combined dataset (Table 2). Unfortunately, further back-mapping analyses to a reference sequence (or the most closely related organism) to identify homologue sequence and/or to validate the accuracy of the different assembly were not conducted due to limited publically available resources. In spite of this, the outcome was consistent with an examination of the completeness of the reference assembly by comparison of the set of core eukaryotic genes (CEG) using the CEGMA as well as the CLC mapping performance (77%–79%) with the final contigs assembled with each assembler (Table 2). While the limited genetic resources in public databases and/or the incompleteness of the three assembly datasets here could contribute to any issues encountered, a cautious interpretation should be made due to a low percentage of complete CEG proteins found in the transcriptome assembly (50%–53%), which could have resulted in low BlastX similarity (30%). In contrast, identification of a large number of unmatched contigs could constitute species-specific sequences present in *Macrobrachium* taxa. While it may be possible to improve the overall assembly by combining use of the three assemblers, only individual assemblies were conducted here.

### 2.3. BLAST Search and Species Distribution

To identify the putative functions of the *M*. *rosenbergii* transcripts, all contig similarity generated with the 20-mer from the CLC assembly were searched against the GenBank non-redundant (nr) database using BlastX applying an *E*-value of < 10^−5^. A total of 13,367 (30.1%) giant freshwater prawn contigs out of 44,407 showed significant similarity (*E*-value < 10^−5^) with sequences in the nr database (Table S1). The remaining 31,040 contigs may represent potential UTRs, non-protein-coding genes, or additional transcripts from giant freshwater prawn-specific genes that are too divergent to be annotated using a similarity search applying the current *E*-value cutoff.

In addition to the high quality transcripts available from the target species, there were transcripts from xenobiotic contaminant species since mRNA in *M*. *rosenbergii* samples came from isolated muscle, ovary, and testis tissues. Therefore, it is possible that some of the sequences identified in our dataset came from xenobiotic species that constitute commensal microorganisms present in *M*. *rosenbergii*. We therefore conducted BlastX analysis and attempted to determine species that showed top matches with *M*. *rosenbergii* sequences. As expected, coding sequences for the majority of contigs (10,025 contigs, 75%) showed significant similarity with contigs (13,367) that matched well to crustacean and other arthropod proteins, a result consistent with previous findings [10,11]. Only a small fraction (4.2%) of our sequences showed their top BlastX match with bacterial sequences, followed by 1.3% with the fungi/metazoan group, and only 0.04% with viral sequences. Further investigation may be warranted to resolve whether non-higher eukaryote hits for *M*. *rosenbergii* sequences with similarity to xenobiotic species may result from symbionts and other microbial taxa present naturally in giant freshwater prawn tissues.

### 2.4. Functional Annotations

**Figure 2:**
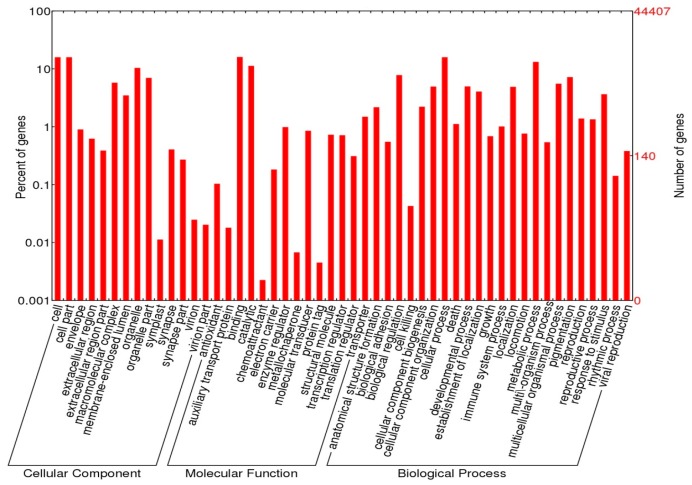
Summary of gene ontology (GO) terms for all combined contigs in *M*. *rosenbergii*. Similarity of contigs generated with the CLC Genomic Workbench were searched and annotated using WEGO.

Gene ontology (GO) terms could be assigned to 44,407 *M*. *rosenbergii* contigs based on BLAST matches to proteins with known functions (Table S1 and Figure 2).

Potential coding sequences were assigned to three different GOs using WEGO—cellular components (15,934 sequences), molecular function (21,436 sequences), and biological processes (34,757 sequences) (Figure 2). InterProScan was used to identify 103,136 protein domains among the 44,407 *M*. *rosenbergii* contigs (Table S1). While not all of the major genes reported in putative KEGG pathways were found here, many of the coding sequences identified here predicted pathways likely to be useful in future investigations that focus on their functions in *M*. *rosenbergii*. From the KEGG analysis, the majority of putative genes were carbohydrate, energy, and amino acid metabolism pathways that are considered as basic metabolism processes (Table S1 and Figure S1). Among non-metabolic pathways, a number of important pathway maps that are potentially involved in sex differentiation and gonadal development were identified. These include: progesterone-mediated oocyte maturation, estrogen signalling, WNT signalling, insulin signalling, GnRH signalling, ErbB signalling, and VEGF signalling (Table S2 and Figure S1). More details of this group of genes are addressed below in a section on “putative genes/proteins potentially affecting sex differentiation and reproductive development and/or function”.

After annotation of *M. rosenbergii* contigs, we conducted further *ab initio* predictions of the potential Open Reading Frames (ORFs) for the 44,407 contigs to identify potential protein-coding genes. A total of 43,833 and 8982 contigs had putative ORFs detected with a minimum length of 10 and 100 amino acids, respectively. The CD-Search tool in NCBI was also used to determine the putative functions of these ORFs and to identify conserved domains, or function units, within the protein query sequences. A total of 2288 (total cluster found), 1239 (total specific features found), and 2453 (total generic features found) ORFs out of 16,164 (total domains found) were identified with conserved domains, suggesting that the ORF-harbouring contigs were derived from functional protein-coding genes as well. Thus, genes mediating reproductive performance await discovery.

### 2.5. Differential Analysis of Gene Expression Profiles in *M. rosenbergii* Ovary and Testis Tissues

**Figure 3:**
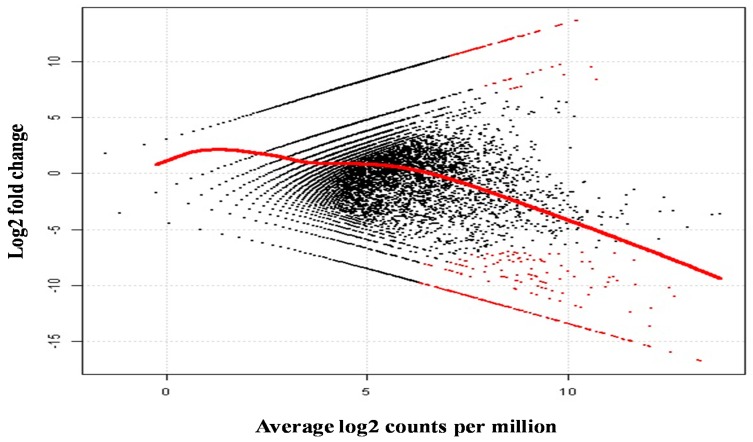
Differential gene expression analysis comparing ovary and testis tissues from *M*. *rosenbergii*. Red dots indicate significantly differentially expressed genes (transcripts or contigs) and the red curved line is average expression strength in the plot.

In the case of tissue specific analysis of differentially regulated and/or expressed genes, numerous genes involved in reproduction, sex differentiation, and development/maturation were identified (Table 3). Differential expression analysis of transcripts demonstrated that most putative genes did not show significant difference in expression patterns between tissues (Figure 3). In the plot of contrast between ovary and testis, the numbers of up- and downregulated genes (contigs) was 112 *vs.* 270, respectively (Table S3). Discovery of these essential genes and their regulatory mechanisms provide new understanding about the complex processes of reproductive development in prawns. Information gained on these genes can have potential applications as molecular markers for *M*. *rosenbergii*. 

### 2.6. Putative Genes/Proteins Affecting Sex Differentiation and Reproductive Development and/or Function

The *M*. *rosenbergii* sequence database was further mined for coding gene and protein sequences potentially involved with sex-differentiation/reproductive-related genes. Despite recent advances in high throughput sequencing technologies, very few genes with such functions have been characterised to date from crustacean species using NGS approaches. From the 44,407 contigs from *M*. *rosenbergii*, a large number of putative genes/proteins were observed with functional characteristics that suggest possible roles in sex-differentiation/reproduction-related functions. Coding sequences with homology to sex-differentiation/reproductive-related genes/proteins have been summarised in Supplementary Tables S1–S4 with contig IDs, sequence descriptions, sequence length, *E*-value, and GOs. In addition, more details of selected KEGG pathway maps with KEGG IDs and contig IDs are accessible in Supplementary Tables S1–S4. A brief explanation for a few selected genes/proteins/pathways is available in the Discussion section.

We identified a number of putative genes, transcription factors, and early gene regulators that are potentially involved in sex differentiation and reproductive development and function in *M*. *rosenbergii*. Further studies need to be performed, however, to understand the specific molecular functions of these reported genes that were highly expressed in both adult prawns compared with expression levels in earlier developmental stages.

### 2.7. Putative Molecular Markers

Of the 44,897 putative SNPs in *M. rosenbergii* contigs identified from alignments of multiple sequences, we observed a much higher number compared with that observed in an earlier 454 FLX run [10,28]. A cautious interpretation should be made because this outcome could result from two different sequencing technologies and/or error rate. A total of 29,043 were putative transitions (*T*_s_) and 15,854 were putative transversions (*T*_v_), giving a mean *T*_s_:*T*_v_ ratio of 1.83:1 across the transcriptome (Table 4 and Table S5). Average SNP frequency was one SNP per 231 bp. The SNP types A ↔ G and C ↔ T were most common, and SNP densities varied among genes. While alignments also identified a total of 8739 indels across the transcriptome, this result must be treated with caution because of technical problems associated with 454 FLX and Ion-Torrent such as reasonable high sequencing error rates (~1%) compared with Illumina platforms (~0.1%). In addition, applying more stringent quality criteria for SNP calling, with a minimum read depth of 10, resulted in a total of 25,602 putative SNPs (16,932 transitions and 8670 transversions) and 4793 putative indels, respectively (data not shown). However, a cautious interpretation should be made because increasing the minimum read depth (*i.e.*, 10) could also increase the chance of losing rare SNPs called from rare transcripts with low read depth. Moreover, a total of 29,184 SSRs or microsatellites comprising 73.44% dinucleotide repeats, 24.61% trinucleotide repeats, and 1.95% tetra-/penta-/hexa-nucleotide repeats were detected (Table 5 and Table S6) in the contigs as well as singletons.

A total of 9040 PCR primer sets were designed to amplify predicted polymorphic SSRs (Table S6), and many putative SNPs were identified. These, however, have yet to be validated as markers with utility for *M*. *rosenbergii* molecular ecological studies because some might be crossing UTR boundaries and un-assembled indels and/or gaps in low coverage regions. In particular, SNPs identified here could simply represent allelic variants, and future studies will be required to validate which ones are real. While applying more stringent quality criteria for variant calling could ensure minimise false positive error rates, it could also lose some positive information. Thus, future readers/users who want to use the current data should do so with caution.

## 3. Discussion

### 3.1. Comparison of Transcriptome Assembly Performance

Here, we extend *de novo* transcriptomes developed for *M*. *rosenbergii* using both 454 FLX and Ion-Torrent platforms. While *de novo* assembly of short reads without a known reference remains a challenging task for non-model species such as *M*. *rosenbergii*, a number of recent studies have demonstrated the utility of transcriptome assembly using Illumina [3,11,29] and Ion-Torrent short read NGS platforms [30,31], in particular, by combining paired-end read sequencing technology to facilitate the assembly.

Considering each assembler utilises different methods for dealing with sequencing errors and single/paired-end information, choosing a suitable assembler and applying optimum parameters is critical to achieving the best possible assembly performance. Assemblers may also differ in their abilities to capture different portions of the transcriptome with accuracy. This is particularly important in transcriptome studies of non-model organisms. In particular, differential gene expression results show variable coverage with transcriptome sequencing, so the choice of a single *k*-mer value usually used with genome assembly cannot generate an assembly that emphasises transcript diversity. The use of multiple assemblers and the application of various *k*-mer lengths while retaining only the best part of each one to form the final assembly output have been demonstrated to be effective for *de novo* transcriptome assembly [25,32]. Thus, we report an efficient assembly and annotation for our *M*. *rosenbergii* transcriptome by applying multiple assemblers and various *k*-mer values to improve assembly sensitivity. Comparisons revealed that assemblies generated with different software programs had associated advantages and disadvantages, particularly with respect to large variation associated with *k*-mer length present among assemblers. Despite this fact, results indicate that Trans-ABySS and Trinity were clearly inferior to CLC for assembly of long transcriptome sequences, indicating the strength of CLC for generating an assembly with better contiguity. In addition, a single type of assembler and/or sequencing platform is not sufficient for the assembly of long and reliable contigs in *de novo* transcriptome sequencing with short read NGS platforms like Illumina or Ion-Torrent [31,33]. While the current combined dataset (454 FLX + Ion-Torrent) was helpful in extending genomic resources of *M*. *rosenbergii*, ongoing improvement in the third-generation sequencing technologies make it possible to overcome all major obstacles that second-generation sequencing currently faces.

According to our previous research on teleost species [30,31], our initial assumption was the current short single-end reads (113 bp) from Ion-Torrent with limited tissue samples might not be enough to improve the quality of *de novo* assembly performance. Thus, the hybrid assembly approach was solely considered from the beginning of this study with a hope that the longer 454 FLX data could be helpful in increasing overall assembly quality. It is speculated that adding a small fraction of 454 FLX data (6%) to the larger part of the Ion-Torrent data (94%) might be helpful in producing better assembly results. In a brief evaluation of quality metrics, the hybrid assembly approach was better than the pure Ion-Torrent assembly (the CLC assembly produced the best result; total contigs 32,800, N50 390 bp, maximum contig length 6071 bp, and average contig length 400 bp). While this comparison strongly supported our initial theory for the assembly strategies, a cautious interpretation should be made for the following reasons: (1) the read length becomes insignificant once it exceeds a certain threshold (the threshold is distinct in different organisms) [34]; and (2) the transcriptome complexity (a large number of alternative splicing events) is in inverse proportion to the quality of *de novo* assembly in all existing *de novo* assemblers [34].

Irrespective of these limitations, adding Ion-Torrent transcriptome data, consisting of short reads with slightly higher sequencing error rates, to the existing 454 FLX data [10] resulted in a larger number of contigs by filling possible gaps and hence by resolving putative chimaeric effects from the 454 FLX data assembly (*i.e.*, isoforms or isotigs). While further lab validation will be required to assess technical artifacts (*i.e.*, error rate), the above outcome could have influenced the generation of the shorter average contig length that resulted from a larger number of contigs. To explain this phenomenon, three possible contributing factors can be considered: (1) the limited number of tissues sequenced here may not have been sufficient to cover a comprehensive transcriptome (or longer average contig length); (2) the single-end reads generated here could not resolve the assembly problem caused by repetitive and/or structural variation regions; and (3) a significant number of polymorphisms were apparent in the samples (18 individuals), while quality differences in raw reads between the two sequencing platforms (*i.e.*, homopolymer quality score) here could have seriously limited the efficacy of extending contig length during the *de novo* assembly. While we pooled 18 samples to increase SNP detection, this approach was a trade-off with *de novo* assembly performance—in particular, the accurate detection of contigs. In order to resolve polymorphism issues in multiple samples and/or multiple sequencing platforms (if SNP detection is not the research objective), a single individual should be used as the resource for generating a reference sequence using third-generation sequencers [35].

While the current data showed a relatively limited number of expressed transcripts (1.7 Gbp) with low *k*-mer lengths (20-mers in CLC), interesting results have been reported in recent transcriptome datasets for *M*. *rosenbergii* generated using the Illumina paired-end sequencing platform [11,12]. In summary, these results suggest that a large number of longer paired-end reads, a larger amount of total sequenced data (*i.e.*, a minimum of 10 Gbp), various tissue samples, and multiple assembly strategies (*i.e.*, multiple *k*-mers) using various assemblers can affect overall the efficiency of *de novo* assembly in terms of contig numbers and average contig length, overcoming pitfalls associated with short reads during *de novo* assembly. In terms of a strategic approach for *de novo* assembly, a recent transcriptome for catfish showed that merging all assemblies from multiple assemblers can provide a more comprehensive and more accurate assembly [25,30].

### 3.2. Annotation Analysis

Quality of the RNA-seq analysis was also confirmed for sequencing depth, GO, and IPR analyses. The distribution of GO terms and various proteins and domains in the IPR analysis generated from higher sequencing depth showed only low similarity with results in previous studies [10,11,12]. This result, however, needs to be treated with caution because the current study did not remove contig redundancy, where multiple contigs matched to the same or a different region of a single gene, which can result mainly from the occurrence of alternative splicing sites, isoforms, and/or incomplete coverage of the gene by a single unigene. For the BLAST analysis here, a substantial number of contigs showed similar putative gene functions in different gene regions (*i.e.*, short reads, partial sequences), resulting in only limited removal of redundant contigs in the CD-HIT-EST. Furthermore, a large number of singletons could consist of either messenger-like non-coding RNA or fragments of untranslated regions of protein-coding genes (or potential sequencing/assembly errors).

Results here also indicate that reported crustacean data are insufficient to cover the whole transcriptome data. The small numbers of crustacean sequences present in the BLAST analyses (Table S1) and the phylum distribution of top-hit species strongly support this view. These results do not indicate that *M*. *rosenbergii* sequences are more similar to sequences from non-crustacean species, but rather reflect the low levels of crustacean sequences available in the public database compared with numbers reported in other non-model species [29,33] including several other crustacean species [2,3,10,29]. Alternatively, it is possible that some may constitute novel genes unique to this species. While information is still only partial, the genes/proteins reported here provide the best available resource published to date for deciphering specific functions involved in molecular processes associated with reproductive traits and reproductive development in *M*. *rosenbergii*. Thus, future studies will be required to understand the molecular functions of specific reported genes.

### 3.3. Putative Genes Associated with Reproductive Traits and Development

Descriptive and quantitative transcriptome analyses could be an important step for interpreting and revealing molecular constituents of the genome, cells, and tissues. The first step toward understanding molecular mechanisms of reproduction is to identify and characterise sex-related genes and regulatory pathways. In the transcriptome of *M*. *rosenbergii* generated here, a large number of important series of transcription factors homologous to known genes related to sex differentiation were identified, including fruitless (Fru), transformer-2 (Tra-2), sex lethal (Sxl), Dmrt-like (doublesex/mab-3 related family), and chromobox genes, which have been well addressed in a number of early crustacean studies [7,36,37,38]. In addition, among the *M*. *rosenbergii* coding sequences identified here, a large number of reproductive-related genes including the kazal-type proteinase inhibitor, the male reproductive-related protein, oocyte-related proteins, sperm-related proteins, testis-related proteins, the vasa-like gene, and vitellogenin were very common and have been well addressed previously in a number of studies where evidence suggests their involvement in the regulation of growth, moulting, and reproduction (mature oocyte, spermatozoa, and gonad) in crustacean [12,39].

Among several pathways selected here, the first interesting pathway involved in sex-related traits was the insulin-signalling pathway. Insulin-like related genes/androgenic gland (AG) insulin-like hormone (IAG) were observed in high frequency in the *M*. *rosenbergii* transcriptome. Evidence for functional roles of IAG has also accumulated in crustaceans and indicates that they have functions as possible regulator/involvements in growth, metamorphosis, immunity, and sex differentiation [40,41,42]. In particular, the identification of AG-specific IAG and genomic sex-related markers offers an opportunity to develop a deeper understanding of the sexual differentiation process in crustaceans and other arthropod species [1,21,22]. While more work will be required in general to understand the influence(s) of IAG on crustacean growth and to elucidate how insulin-like peptides regulate sexual shifts (e.g., for appropriate intervention) in crustaceans because growth is often sexually dimorphic in a wide array of malacostracans [43,44], a recent study has suggested that sexual differentiation in crustaceans involve more than a single *M*. *rosenbergii*–IAG receptor, highlighting the complexity of sexual differentiation and maintenance [4].

Other interesting pathways involved in oogenesis, spermatogenesis, and gonadal maturation were VEGF/ErbB/GnRH/Wnt/MAPK signalling pathways. The cell division cycle 2 (Cdc2) kinase identified in the VEGF signalling pathway, a catalytic subunit of maturation-promoting factor and a central factor for inducing meiotic maturation of oocytes, has been reported to play an essential role in inducing germinal vesicle breakdown and for generating the meiotic apparatus during crab oocyte maturation in the Chinese mitten crab, *Eriocheir sinensis* [45]. In a number of crustacean studies, expression of cyclin A, B, and Cdc2 mRNA, identified in ErbB/GnRH signalling pathways, is related to oogonial proliferation (mitosis), oocyte meiosis, ovarian development, and oocyte maturation [45,46,47]. The extracellular signal-regulated kinase 2 (Erk2) (also termed ERK, mitogen-activated protein kinases (MAPK), or MAPK1), identified in most major pathways addressed above, an important factor in the ERK signal transduction pathway and in regulation mechanisms during oocyte development and ovarian pathology, has been observed to be involved in ovarian development in green mud crab, *Scylla paramamosain* [48].

The ubiquitin-conjugating enzyme E2r (UBE2r) (also called UBC3 or CDC34), identified in most major pathways addressed above except VEGF signalling pathway, is one of the conjugating enzymes in the UPP system that influences cAMP-inducible gene regulation during both meiotic and mitotic cell cycles. This gene has been reported to be differentially expressed at different stages in the developing ovary and testis, which suggests that UBE2R could have an important role in oogenesis and spermatogenesis [49]. In the case of MnUbc9, an important conjunction enzyme in the small ubiquitin-like modifier (SUMO) pathway, differential expression of MnUbc9 in the embryo and ovary implies that MnUbc9 could play an important role in embryogenesis and oogenesis in *M*. *nipponense* [50]. The ubiquitin (UB)/proteasome pathway plays an essential role in gametogenesis, and, additionally, the fusion protein is the precursor for mono-ubiquitin [51]. In eukaryotes, UB moiety, which can fuse to either ribosomal polypeptide L40 or S27, acts as a chaperone in ribosomal biogenesis prior to proteolytic separation from L40 and S27, suggesting an involvement in cell growth and cell division [52,53]. *In situ* hybridization of UBS27 and UBL40 in testis and ovary showed that they play key roles in gametogenesis and affect reproductive success in *E*. *sinensis* [54].

Adding to the major pathways addressed here, a number of key genes/proteins have also been reported as essential and/or possible roles for the regulatory mechanisms governing reproductive processes in crustacean taxa: namely, the broad-complex protein (Br-c) (a possible important role during ovarian development in *P*. *monodon*) [55], cathepsin related genes (a possible involvement in the final stages of oocyte maturation in *Marsupenaeus japonicas*) [56], the KIFC1-like kinesin gene (a critical function in spermiogenesis in *E*. *sinensis*) [57], myosin va (a possible involvement in acrosome biogenesis and nuclear morphogenesis during spermatogenesis in *E*. *sinensis*) [58], the protein phosphorylatin (a potential role in regulating cell cycle and in ovary development in *P*. *monodon*) [59], and Scygonadin (Scy) (a potential role in in fertilization and reproduction in both male and female crabs during mating) [60].

While, to date, there have been few studies of gene expression patterns in *M*. *rosenbergii*, some recent studies have investigated reproductive regulation and sex-differentiation mechanisms [2,3]. Here, we have identified a number of putative genes, proteins, transcription factors, and early regulators that are potentially involved in gonad development and associated functions in *M*. *rosenbergii*. In addition, numerous genes/enzymes were identified in our selected KEGG pathway maps (Table S2 and Figure S1). While no further lab validation was undertaken, information identified here could lead to a greater understanding of important signalling pathways, especially with respect to fertilization, sexual differentiation, maturation, and maintenance of sexual characteristics that will facilitate future research on the reproductive biology of prawns [29,42,61]. In time, these could potentially yield key biomarkers for the gonad development and molecular function of sexual characteristics that currently remain largely unknown in *M*. *rosenbergii*. Additional transcription factors in this series are likely to be identified when more transcriptomes are developed from multiple tissues, and early developmental stages are investigated. Furthermore, gonadal tissues are likely to provide a great source of sex-biased gene expression profiles in future *Macrobrachium* studies. Thus, characterization of these genes in *M*. *rosenbergii* could provide a solid foundation for understanding the physiological role that these genes play in sex differentiation in crustaceans more widely. Additionally, the large number of SNPs and SSRs resolved in the current study provide a quantum increase in EST-derived SNPs and SSRs in crustaceans, demonstrating the high efficiency of high-throughput transcriptome analysis for developing SNP and SSR markers. While markers identified here will need verification before they can be used in molecular applications, a number of sex-specific and reproductive-related markers can be designed that could play roles in rapid and low-cost gender identification at early developmental stages and in the early maturation evaluation of *M*. *rosenbergii*.

### 3.4. Limitations of the Current Study

While the current study was constrained somewhat in research scope (see below), it does engender suggestions for improving the transcriptomic analysis of *M*. *rosenbergii* in the future, namely:

(i) Limited samples: Prawns (only three tissue sample types) were only collected at final harvest, which resulted in limited information on gonadal development in both sexes without associated histological studies (developmental stages during vitellogenesis and spermatogenesis). Future experiments should be designed to include a wider range of tissues and include multiple stages of gonadal development in both sexes for fine scale gene expression. This approach would likely result in greater differential gene expression between the sexes as well as identify key genes/proteins that are involved in crustacean reproduction.
(ii) A small and single-end read transcriptome dataset: A minimum of 10 Gbp with longer and/or paired-end reads with multiple tissue samples from a range of developmental stages should be used to generate a comprehensive reference transcriptome because different types of reads to assemble a *de novo* transcriptome (differing in both length and pairing attributes) might have substantial influence on transcriptome downstream analyses/results. More importantly, the effect of paired-end *vs.* single-end strategy has been reported to have a much greater impact in terms of false positives than sequencing length [62]. Thus, the paired-end approach could be an ideal strategy to significantly improve the tracking of alternate splice junctions, indels, and differential gene expression [63]. In addition, applying multiple assembly strategies (*i.e.*, multiple *k*-mers) using various assemblers can affect overall efficiency of *de novo* assembly in terms of contig numbers and average contig length, overcoming pitfalls associated with short reads during *de novo* assembly. In particular, merging all assemblies from multiple assemblers using CD-HIT-EST and/or EvidentialGene tr2aacds pipelines could provide a more comprehensive and more accurate assembly [25,30].
(iii) Requirement for lab validation: The support from substantial wet lab validation is required to strengthen the quality of data. This approach was not, however, available in the current study, so we recommend the validation of candidate genes (*i.e.*, reproductive-related genes), SSRs, and SNPs in the future. In particular, correlations between developmental stages and gene expression profiles validated using a qRT-PCR approach would allow inferences to be made about specific gene functions related to prawn reproduction.

## 4. Materials and Methods

### 4.1. Tissue Material and RNA Extraction

A total of 18 mature *M*. *rosenbergii* individuals at harvest, reared in a prawn stock improvement program in, Bac Ninh (Research Institute for Aquaculture No. 2 (RIA2)), Vietnam [10,15], were used for the current study. Muscle and ovary tissues were sampled from 9 adult females (average body weight 31 g), and testis tissue was sampled from 9 adult males (average body weight 50 g, three blue claw (81 g), 3 orange claw (57 g), and 3 small (12 g), respectively). RNA extraction and quality check processes were consistent with our previous work (see for 454 FLX data) [10] before using the same mRNA samples for Ion-Torrent sequencing. High-quality mRNAs from pooled muscle, ovary, and testis tissue from males and females were: (1) fragmented into 200-bp fragments using the Ion Total RNA-Seq Kit (Life Technologies, Carlsbad, CA, USA); (2) cleaned with RiboMinus Concentration Module (Invitrogen, Carlsbad, CA, USA); and then (3) converted to cDNA using Ion Total RNA-Seq kit (Life Technologies). A Qubit 2.0 fluorometer (Invitrogen) and a Bioanalyzer (Agilent, Santa Clara, CA, USA) were used to quantify cDNA yields. Templates were prepared using the OneTouch Ion^TM^ Template Kit (Life Technologies), and successful templates (each tissue library) were sequenced on 316 semiconductor chips (four chips; 1 muscle, 2 ovary, and 1 testis libraries) using the Ion PGM^TM^ 200 Sequencing Kit and PGM chemistry (Life Technologies) for Ion-Torrent sequencing.

### 4.2. Sequence Cleaning and Assembly

Raw single-end short read transcript data generated from 454 FLX were pre-processed to remove nonsense sequences including adapters, primers, very short (<50 bp) sequences, ambiguous nucleotides (‘N’ at the end of reads) and low-quality sequences using the NGS QC ToolKit software [64]. All raw sequence reads generated from the PGM sequencer were run through the automatic trimmed sequences applying default filtering parameters on the Ion-Torrent server to remove sequencing adapters, very short sequences (<30 bp), and low-quality sequences (*Q* < 20). Sequence reads collected from both the 454 FLX and Ion-Torrent were then converted to FastQ files and further assessed for quality (*Q* > 20). In order to obtain a comprehensive and reliable assembly, three different assemblers, namely, CLC Genomic Workbench (v6.5), Trans-ABySS (v1.4.4) [65], and Trinity (r2013-02-16) [24], were used for *de novo* assembly. On the assumption that some transcripts would be replicated among tissue datasets, trimmed sequences from the three libraries were also merged in a combined single dataset from both platforms (454 and Ion-Torrent) and assembled using *de novo* assembly. In brief, the CLC *de novo* assembly was performed with multiple *k*-mer lengths based on input data from default (*k* = 20) to maximum (*k* = 60) settings, with a 10-mer difference in each setting using a minimum contig length of 200 bp. In the cases of the Trans-ABySS and Trinity assemblies, a multiple *k*-mer approach with every 10-mer value from 25 to 95 for Trans-ABySS and a default 25-mer value for Trinity was used so as to maximise assembly contiguity and sensitivity. Following this, the best assembly setting selected from the merged dataset trial (number of contigs, N50 length, average contigs length, and maximum contig length) was applied to each library dataset of mRNA sequences from muscle, ovary, and testis tissue libraries because each was considered to be representative of the transcriptome of that tissue type at the time of sampling. While some identical contigs (or partial contigs) were generated from more than a single assembly or library introducing duplicates, additional redundancy removal was not conducted here in order to maximise total contig numbers. In the CD-HIT search [66], approximately 150–950 duplication contigs were identified and/or clustered, so we decided to use the original contigs for all further downstream analyses. Quality of the assembly was assessed using the CEGMA pipeline to assess transcriptome assembly completeness by identifying the presence of 248 highly conserved core proteins that are found in a wide range of eukaryotes [67]. Clean read mapping was performed using CLC software with the process of aligning short reads to a reference sequence (the final contigs assembled with each assembler). Additional misassembly assessments (allelic variants, alternative splicing/isoforms, and chimaeric effects) were not conducted here.

### 4.3. Annotation of mRNAs

BlastX searches of the NCBI GenBank non-redundant (nr) database were performed only on contigs to identify putative mRNA functions (*E*-value threshold < 10^−5^). The Blast2GO was used (1) to predict functions of contigs; (2) to assign Gene Ontology terms; (3) to predict metabolic pathways using Kyoto Encyclopaedia of Genes and Genome (KEGG) [68]; and (4) to identify protein domains using the InterProScan tool [69]. The number of contigs annotated with each GO term from each library was quantified using WEGO [70]. The orfPredictor program was also used to predict ORFs for all contigs obtained after the assembly because of the low number of sequences for non-model species in public databases [71]. ORFs were identified in both strand orientations considering all six frame combinations, and transcript sequences were determined by BlastX alignment if applicable. The CD-Search tool in NCBI was also used to determine the putative functions of ORFs and to identify conserved domains, or functional units, within the protein query sequences [72].

### 4.4. Expression Analysis

Reads from two tissues (ovary and testis) were mapped back to contigs generated from CLC assembly (default parameters). Since the muscle library was only available from the female library, it was not included in further expression analyses. Expression level was calculated using the number of aligned reads to contigs using RobiNA (v1.2.4) [73] applying default parameter settings. Normalization and statistical evaluation of differential gene expression was performed using edgeR [74] with the Benjamini–Hochberg method for multiple testing corrections (*p*-value < 0.01). The raw data were normalised according to the default procedure in the differential expression analysis package (log-fold change min = 1 and estimate reads per kilobase per million reads (RPKM) expression measure values), and dispersion was estimated using the auto setting with RobiNA software.

### 4.5. Identification of SSR Motifs and SNPs

The QDD program was used to screen SSR motifs [75]. Default settings, a minimum of 6 repeats for di-nucleotide SSRs, and a minimum of 5 repeats for all other SSR types were employed to detect perfect and compound motifs (from di-nucleotide to hexa-nucleotide motifs). The maximum interruption between 2 neighbouring SSRs to be considered a compound SSR was assigned at 100 nucleotides. The Primer3 was used to design PCR primers, flanking each unique SSR region identified [76].

For putative SNP detection, sequencing reads were mapped onto assembled contigs. While default parameters were applied to identify the final set of potential SNPs, a base quality score of >20 was set to assess the quality of reads at a position for SNP detection using Burrows-Wheeler Aligner (BWA) and Samtools [77]. SNPs, variations compared with the consensus sequence, were counted under the criteria of a read depth of 4 and minimum variant frequency of 2 or more. Indels were segregated into simple types containing an insertion or deletion of at least a single nucleotide compared with the reference sequence or complex types also containing nucleotide substitutions. While the current SNP calling/filtering criteria were processed to ensure the high quality SNP detection, it should be noted that remaining contaminants caused by sequencing platform artifacts (*i.e.*, sequencing error) can still inflate the predicted/estimated SNP errors. Thus, additional depth coverage information of “Supplementary Table S5” can provide more useful SNP information for each predicted/estimated SNP (Column F: Total Depth). Total number of transitions *vs.* transversions (*T*_s_/*T*_v_) and an overall ratio were calculated across the dataset.

## 5. Conclusions

Here, we have confirmed that short read sequence data can be efficient for characterising transcriptomes of a non-model prawn species using *de novo* assembly of combined 454 FLX and Ion-Torrent data. While outcomes are strongly influenced by the assembly methods and assemblers used, sequence quality, and expressed transcript numbers, the strategy for *de novo* assembly described here has wider application in other species. Low levels of transcriptomic and genomic data in public databases to date has limited our understanding of the molecular mechanisms that influence reproductive traits and sex differentiation in *M*. *rosenbergii*. The 44,407 contigs identified here represent a major transcriptomic resource for *M. rosenbergii* and will also be useful for functional genomics studies of other *Macrobrachium* species and be relevant for evolutionary studies. The significant number of putative reproductive-related transcripts identified here should facilitate genomics approaches for manipulating reproductive regulatory mechanisms in domesticated *M. rosenbergii* culture stocks. In addition, the numerous putative SNPs and SSRs identified provide valuable targets for identifying polymorphisms in *M*. *rosenbergii* populations useful for population genomic studies. It is anticipated that the results from this study in the future can contribute significantly towards assembly and annotation of the complete giant freshwater prawn genome. Such resources will likely also be essential for comparative genomics studies (structural and functional annotation) in other prawns and related crustacean species as well.

## Figures and Tables

**Table 1:** Overview of sequencing reads and reads after processing.

Statistics	454 FLX				Ion-Torrent			
Dataset Name	All	Muscle	Ovary	Testis	All	Muscle	Ovary ^d^	Testis
TNB before processing (Mbp) ^a^	244.37	114.38	86.06	43.94	1475.49	302.71	751.09	421.69
Total number of reads	787,731	367,379	279,393	140,959	12,945,479	3,027,258	6,477,802	3,440,419
Average read length (bp)	310	311	308	311	113	100	116	123
GC percentage (%)	48.30	50.45	44.88	49.40	42.26	48.60	47.10	46.70
TNB after trimming and processing (Mbp) ^a^	234.03	109.88	81.90	42.25	1269.81	252.13	644.89	374.80
TNR used for assembly ^b^	754,374 (95.77%)	352,936 (96.07%)	265,907 (95.17%)	135,531 (96.15%)	11,141,087 (86.06%)	2,521,259 (83.29%)	5,561,961 (85.86%)	3,057,867 (88.88%)
ARL after trimming and processing (bp)	261	267	258	258	94	87	95	100
GC percentage (%) ^c^	48.60	49.50	44.40	49.60	42.90	47.10	38.90	46.70

^a^ Total number of bases (TNB); ^b^ Total number of reads (TNR); ^c^ Average read length (ARL); ^d^ Two 316 Chips run. Trimming and processing indicate Q20 and 50 bp for 454 FLX and 30 bp for Ion-Torrent.

**Table 2:** Assembly results with best *k*-mer setting from the *M*. *rosenbergii* transcriptome 454 FLX and Ion-Torrent datasets using different *de novo* assemblers.

Assembler	Parameter	All	Muscle	Ovary	Testis
Trans-ABySS (*K* = 75)	Total base of reads (bp)	1,294,937,751	317,222,362	603,806,324	343,353,395
	No. of total contigs	40,225	4556	25,482	5335
	Total bases of contigs (bp)	13,707,795	1,629,728	8,896,935	1,788,051
	No. of contig ≥ 1000 bp	366	83	284	51
	Contig N50 (bp)	348	357	357	333
	Mean contig length (bp)	341	358	349	335
	Largest contig (bp)	2750	4194	2387	2054
	^†^ CEGMA/Mapping (%)	50.11/77.71			
Trinity (*K* = 25)	Total base of reads (bp)	1,294,937,751	317,222,362	603,806,324	343,353,395
	No. of total contigs	78,007	11,337	70,352	17,260
	Total bases of contigs (bp)	27,946,414	4,152,262	23,845,796	5,580,093
	No. of contig ≥ 1000 bp	912	182	439	99
	Contig N50 (bp)	368	370	344	322
	Mean contig length (bp)	358	366	339	323
	Largest contig (bp)	4480	3223	3350	2694
	^†^ CEGMA/Mapping (%)	51.81/77.27			
CLC Bio (*K* = 20)	Total base of reads (bp)	1,294,937,751	317,222,362	603,806,324	343,353,395
	No. of total contigs	44,407	8397	35,847	10,604
	Total bases of contigs (bp)	19,415,235	3,826,821	15,013,223	4,260,690
	No. of contig ≥ 1000 bp	9259	1871	6860	1770
	Contig N50 (bp)	438	448	417	399
	Mean contig length (bp)	437	456	419	402
	Largest contig (bp)	9495	8037	5139	3978
	^†^ CEGMA/Mapping (%)	53.09/79.26			

^†^ CEGMA (Core Eukaryotic Genes Mapping Approach) and CLC Genomic Workbench Mapping.

**Table 3:** Top 20 differently expressed genes from ovary and testis tissues in *M*. *rosenbergii*. Minimum *E*-value (<10^−5^) and exclude hypothetical/predicted proteins.

Contig Number	Putative Function	Length (bp)	*E*-Value	Log2 Fold Change	Average Log2 Counts Per Million	*p*-Value	False Discovery Rate
**30,784**	kazal-type protease inhibitor	976	4.82 × 10^−85^	−16.66	13.23	6.17 × 10^−10^	1.30 × 10^−05^
**30,770**	kazal-type proteinase inhibitor	631	2.18 × 10^−22^	−14.96	11.53	1.19 × 10^−08^	4.68 × 10^−05^
**30,724**	male reproductive-related protein a	629	1.12 × 10^−16^	−15.43	12.00	5.23 × 10^−09^	3.67 × 10^−05^
**30,822**		342	3.20 × 10^−24^	−13.97	10.54	6.58 × 10^−08^	0.000104
**30,728**		432	2.54 × 10^−08^	−13.97	10.5	6.60 × 10^−08^	0.000104
**30,725**		281	1.49 × 10^−23^	−13.71	10.28	1.03 × 10^−07^	0.000129
**30,740**		369	6.13 × 10^−08^	−12.50	9.06	8.37 × 10^−07^	0.00046
**30,846**	metalloproteinase-like	1210	3.65 × 10^−52^	−15.01	11.58	1.08 × 10^−08^	4.68 × 10^−05^
**30,807**		511	2.19 × 10^−32^	−13.91	10.48	7.24 × 10^−08^	0.000109
**30,868**	periaxin-like protein	1872	4.31 × 10^−08^	−14.57	11.14	2.30 × 10^−08^	6.46 × 10^−05^
**30,726**	cysteine-rich motor neuron 1	742	1.09 × 10^−07^	−14.52	11.09	2.54 × 10^−08^	6.66 × 10^−05^
**30,757**	male reproductive-related protein mar-mrr	254	2.48 × 10^−08^	−14.33	10.90	3.54 × 10^−08^	8.06 × 10^−05^
**30,716**		272	1.26 × 10^−42^	−14.22	10.79	4.28 × 10^−08^	8.18 × 10−^05^
**30,718**		280	9.61 × 10^−43^	−12.58	9.14	7.33 × 10^−07^	0.000428
**31,029**	blastula protease-10	1528	1.22 × 10^−34^	−13.98	10.55	6.45 × 10^−08^	0.000104
**19,861**	matrix metalloproteinase-9	1103	7.60 × 10^−20^	−13.88	10.45	7.65 × 10^−08^	0.000111
**30,737**	keratin associated protein	771	4.77 × 10^−19^	−13.76	10.33	9.47 × 10^−08^	0.000126
**30,785**	3d domain protein	510	6.23 × 10^−06^	−13.67	10.24	1.10 × 10^−07^	0.000129
**30,768**	lpxtg-motif cell wall anchor domain protein	1231	5.25 × 10^−23^	−13.65	10.22	1.13 × 10^−07^	0.000129
**30,916**	serine proteinase inhibitor	324	1.44 × 10^−16^	−13.63	10.20	1.18 × 10^−07^	0.00013
**30,796**	insulin-like androgenic gland factor	614	1.13 × 10^−110^	−13.45	10.01	1.62 × 10^−07^	0.000159
**30,954**	von willebrand factor d and egf domain-containing	1632	1.12 × 10^−27^	−13.36	9.93	1.87 × 10^−07^	0.000175
**32,194**	apolipoprotein d-like	513	5.53 × 10^−08^	−13.12	9.69	2.85 × 10^−07^	0.000239
**31,036**	hemolectin cg7002-pa	2221	8.78 × 10^−25^	−13.05	9.62	3.21 × 10^−07^	0.000252
**30,739**	epididymal sperm-binding protein 1-like	1043	3.63 × 10^−16^	−12.63	9.20	6.67 × 10^−07^	0.000412
**30,881**	2 RNA ligase family protein	364	3.61 × 10^−06^	−12.50	9.06	8.41 × 10^−07^	0.00046
**30,750**	keratin associated protein	668	3.21 × 10^−11^	−12.49	9.05	8.52 × 10^−07^	0.00046
**30,749**	fibronectin 1b	677	1.57 × 10^−06^	−12.41	8.97	9.84 × 10^−07^	0.00052

**Table 4:** Summary of putative single nucleotide polymorphism (SNP) and Indel distribution.

**All SNPs**	**Transitions**				**Transversions**								
	**(29,043 (64.69%))**				**(15,854 (35.31%))**								
	**CT**	**GA**	**AG**	**TC**	**AC**	**GT**	**GC**	**AT**	**TG**		**CA**	**CG**	**TA**
44,897	7290	7421	7249	7083	2046	2024	1304	2445	2070		1905	1335	2725
100 (%)	16.2	16.5	16.1	15.7	4.5	4.5	2.9	5.4	4.6		4.2	2.9	6.0
**All Indels**	**Simple**				**Complex**								
	**(8490 (97.15%))**				**(249 (2.85%))**								
	**Insertion**		**Deletion**		**Insertion**					**Deletion**			
8739	4369		4121		207					42			
100 (%)	49.99		47.16		2.37					0.48			

**Table 5:** Summary of putative simple sequence repeat (SSR) nucleotide classes among different nucleotide types. Both contig and singleton sequences were used to predict SSR loci.

SSR Types	Total		Contigs		Singletons	
	Discovered Motifs	Designed Primers	Discovered Motifs	Designed Primers	Discovered Motifs	Designed Primers
Di-nucleotide	21,433	6643	3754	977	17,679	5666
AT/TA	7598	2414	1323	358	6275	2056
CA/AC	5775	1752	1018	259	4757	1493
CG/GC	455	133	50	11	405	122
CT/TC	0	0	0	0	0	0
GA/AG	7605	2344	1363	349	6242	1995
GT/TG	0	0	0	0	0	0
Tri-nucleotide	7181	2218	1432	375	5749	1843
AAC/ACA/CAA	538	174	80	20	458	154
ACG/CGA/GAC	199	55	59	13	140	42
ACT/CTA/TAC	385	120	64	19	321	101
AGC/GCA/CAG	750	224	225	60	525	164
AGG/GAG/GGA	631	181	228	52	403	129
AGT/GTA/TAG	0	0	0	0	0	0
CAT/ATC/TCA	1327	402	228	53	1099	349
CCA/CAC/ACC	174	49	97	26	77	23
CCG/CGC/GCC	40	13	27	7	13	6
CCT/CTC/TCC	0	0	0	0	0	0
CGG/GGC/GCG	0	0	0	0	0	0
CTG/TGC/GCT	0	0	0	0	0	0
CTT/TTC/TCT	0	0	0	0	0	0
GAA/AAG/AGA	1616	513	215	61	1401	452
GAT/ATG/TGA	0	0	0	0	0	0
GTT/TGT/TTG	0	0	0	0	0	0
TAA/ATA/AAT	1521	487	209	64	1312	423
TCG/CGT/GTC	0	0	0	0	0	0
TGG/GTG/GGT	0	0	0	0	0	0
TTA/TAT/ATT	0	0	0	0	0	0
≥Tetra-nucleotide	570	179	52	13	518	166

## Supplementary Materials

Supplementary materials can be found at http://www.mdpi.com/1422-0067/17/5/690/s1.
