# Supplementary material for: Optimizing Hybrid de Novo Transcriptome Assembly and Extending Genomic Resources for Giant Freshwater Prawns (Macrobrachium rosenbergii): The Identification of Genes and Markers Associated with Reproduction
Source: Int J Mol Sci. 2016 May 7;17(5):690. doi: 10.3390/ijms17050690 (PMC4881516; doi:10.3390/ijms17050690)
Supplement: Supplementary file 1 [file ijms-17-00690-s001.zip › ijms-125344-Supplementary Materials/ijms-125344-Supplementary Figure.pdf]

Supplementary Materials: Optimizing Hybrid *de Novo* Transcriptome Assembly and Extending Genomic Resources for a Giant Freshwater Prawn (*Macrobrachium rosenbergii*): The Identification of Genes and Markers Associated with Reproduction

Hyungtaek Jung, Byung-Ha Yoon, Woo-Jin Kim, Dong-Wook Kim, David A. Hurwood, Russell E. Lyons, Krishna R. Salin, Heui-Soo Kim, Ilseon Baek, Vincent Chand and Peter B. Mather

Table S1. Table summary of Blast2Go results including Gene Ontology, KEGG, InterProScan domain search for all combined contigs in *M. rosenbergii*. Similarity of contigs generated with the CLC Genomic Workbench were searched and annotated using BlastX against the non-redundant database in NCBI with an *E*-value cutoff of 1×10^{-5}.

Table S2. Summary of KEGG IDs for all combined contigs in *M. rosenbergii*. Similarity of contigs generated with the CLC Genomic Workbench were searched and annotated using KAAS.

Table S3. Differentially expressed genes between ovary and testis tissues for all combined *M. rosenbergii* contig sequences. Contigs generated with the CLC Genomic Workbench were employed as a reference sequence.

Table S4. Gene lists potentially involved in reproductive-related development and sex differentiation for all combined *M. rosenbergii* contig sequences. Contigs generated with the CLC Genomic Workbench were employed to identify contigs.

Table S5. Putative SNPs for all combined *M. rosenbergii* contig sequences. Contigs generated with the CLC Genomic Workbench were employed as a reference sequence to identify polymorphisms.

Table S6. Putative microsatellite loci for all combined *M. rosenbergii* contig sequences. Contigs generated with the CLC Genomic Workbench were employed as a reference sequence to identify microsatellite motifs.

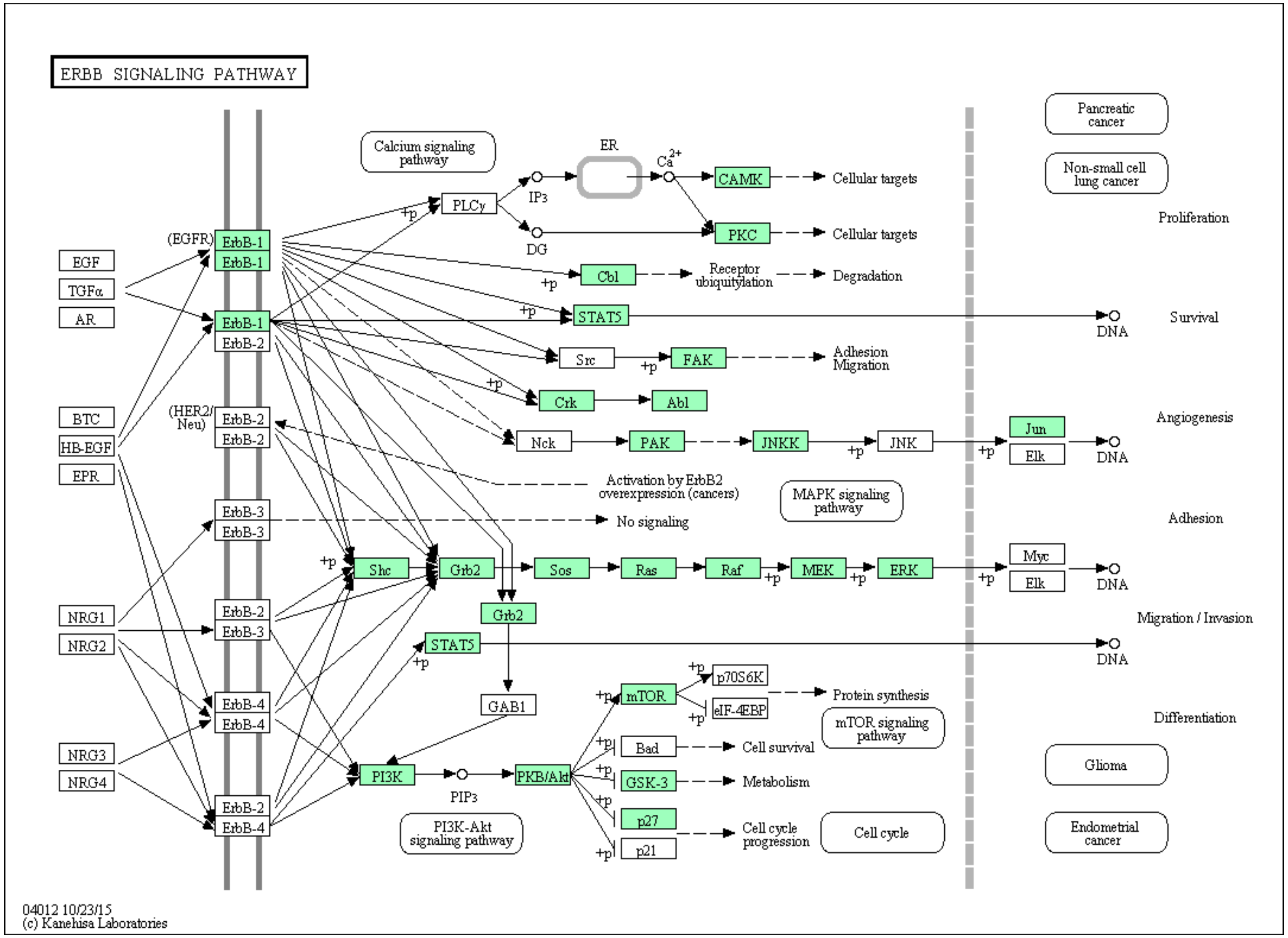

(**A**)

Figure S1. *Cont.*

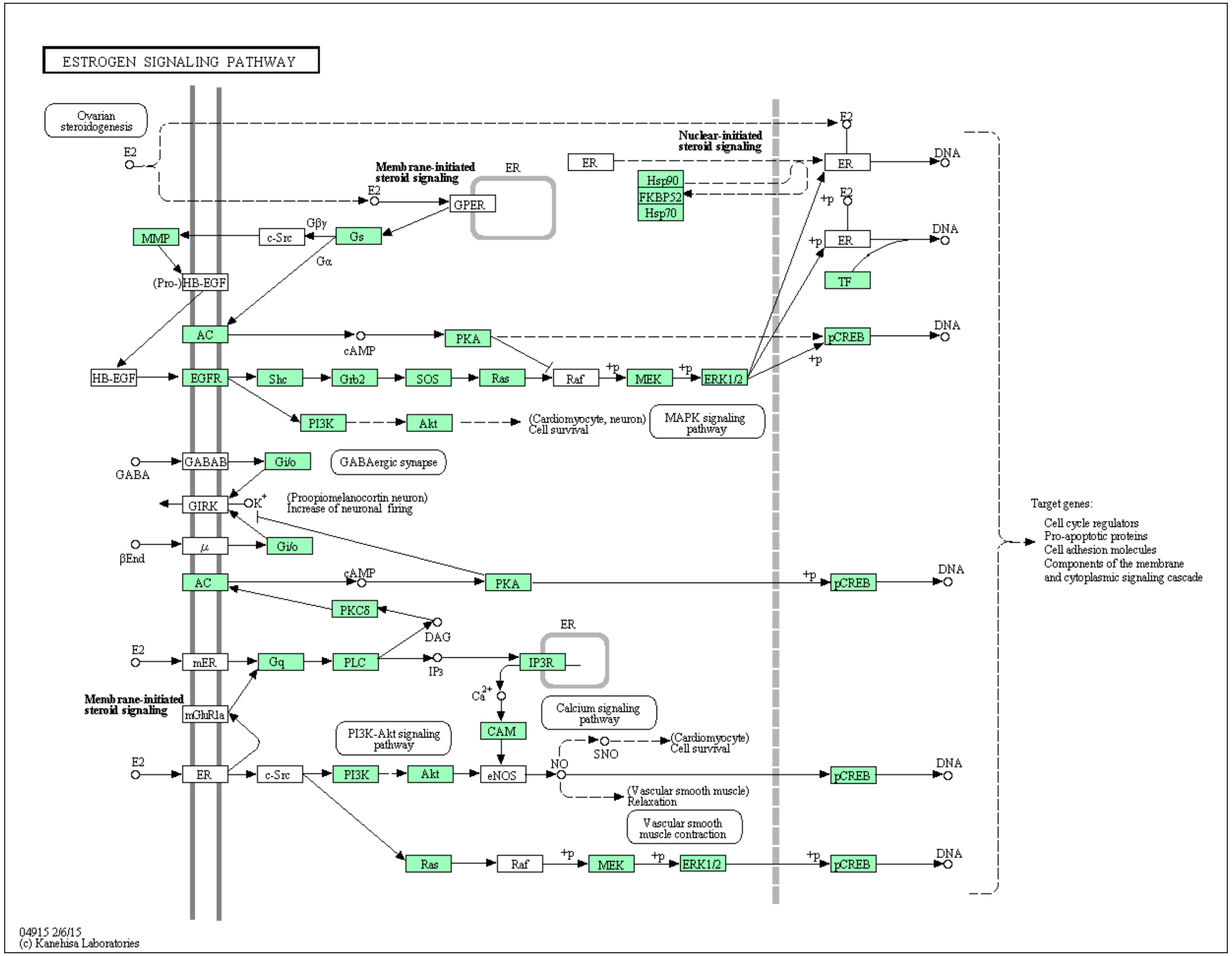

(B)

Figure S1. *Cont.*

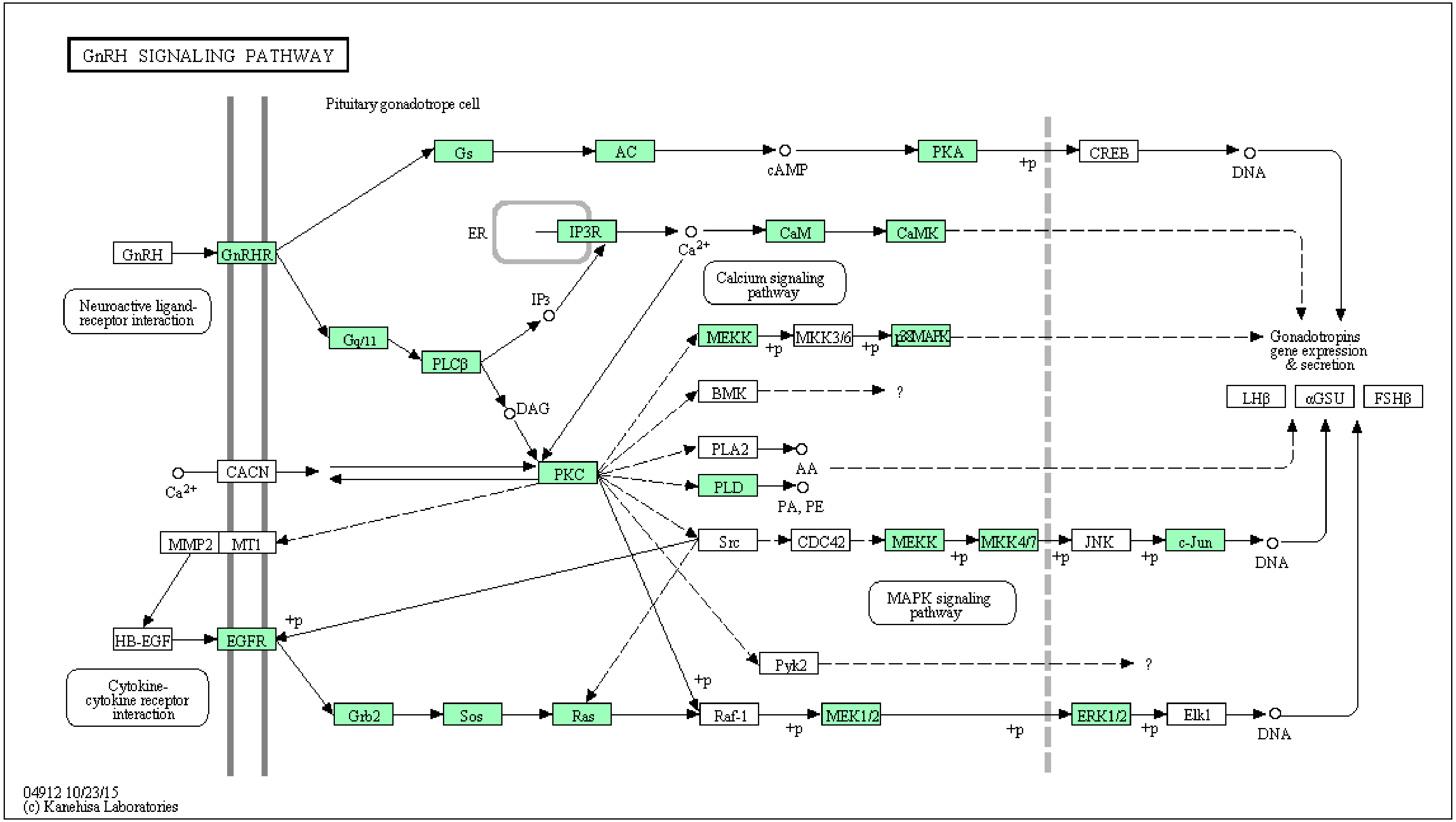

(C)

Figure S1. *Cont.*

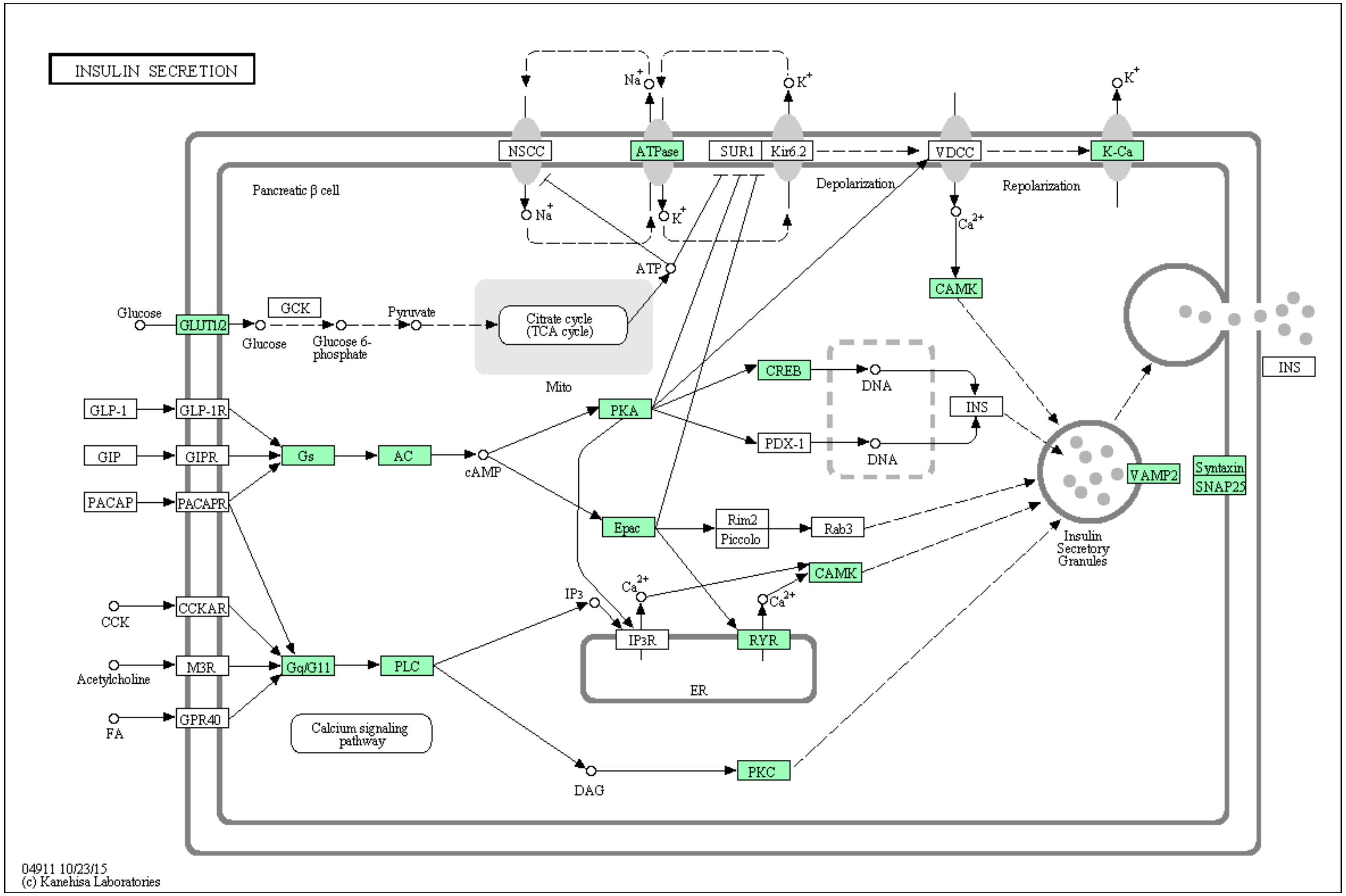

(**D**)

Figure S1. *Cont.*

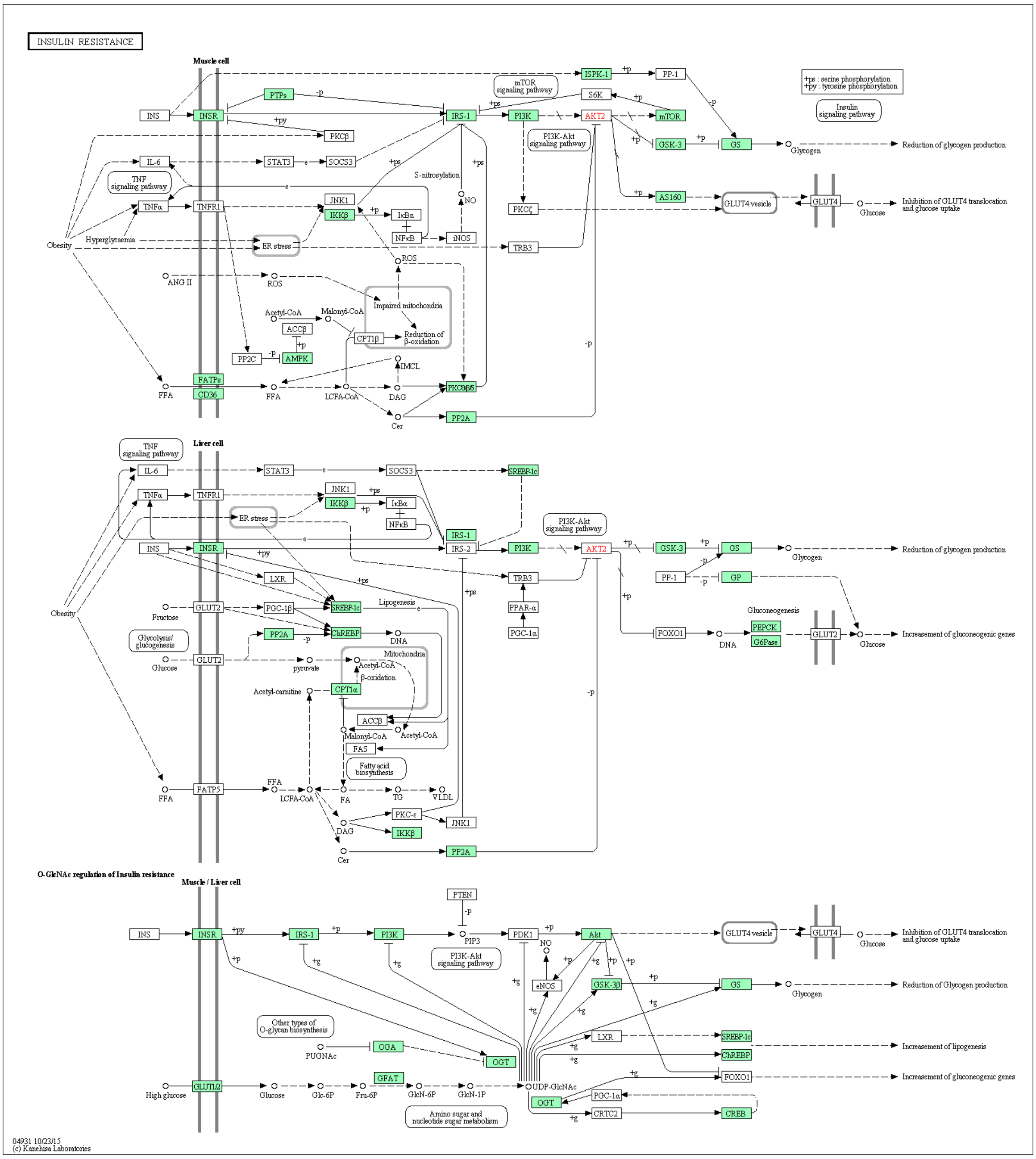

(**E**)

Figure S1. *Cont.*

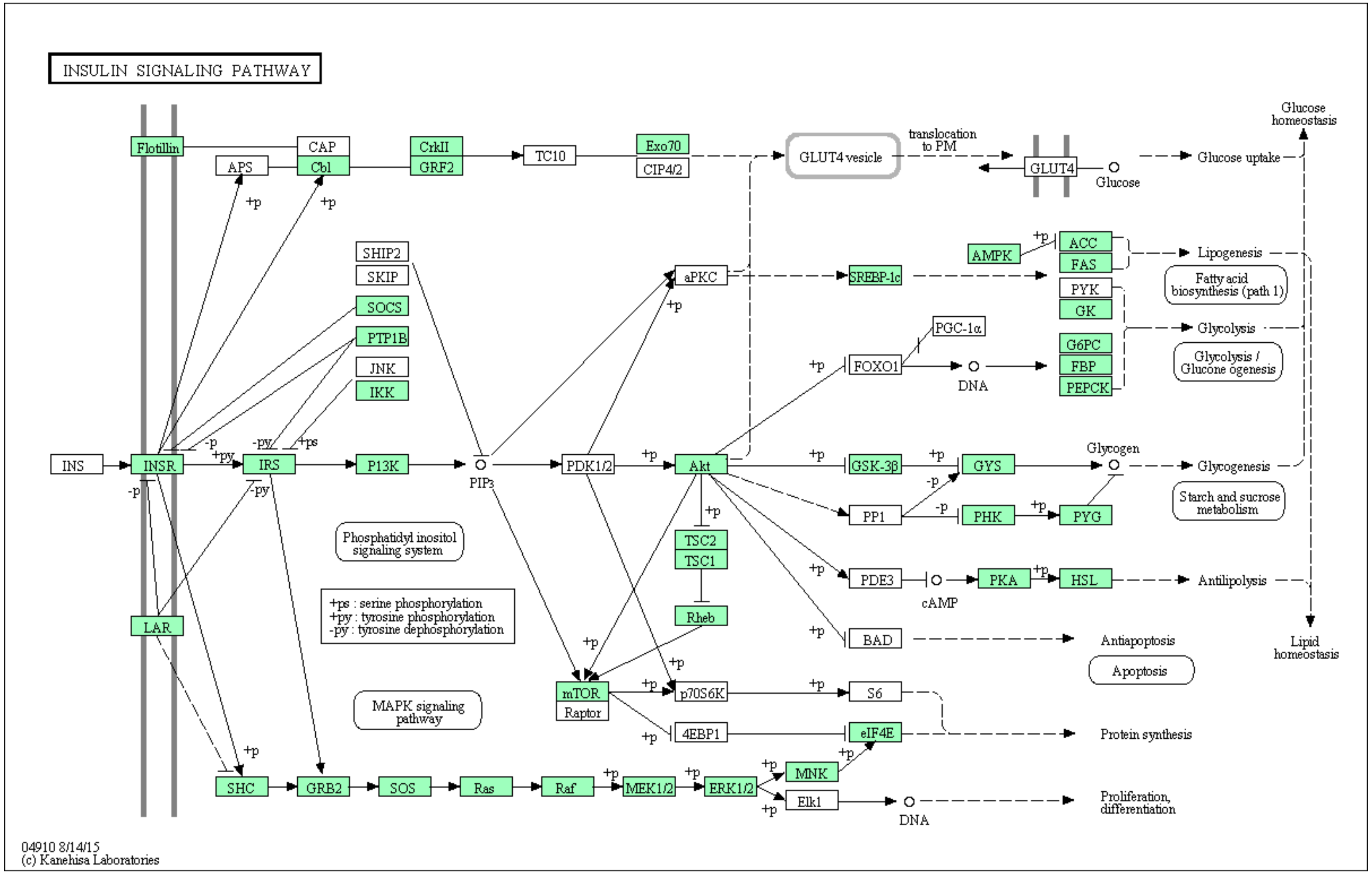

(F)

Figure S1. *Cont.*

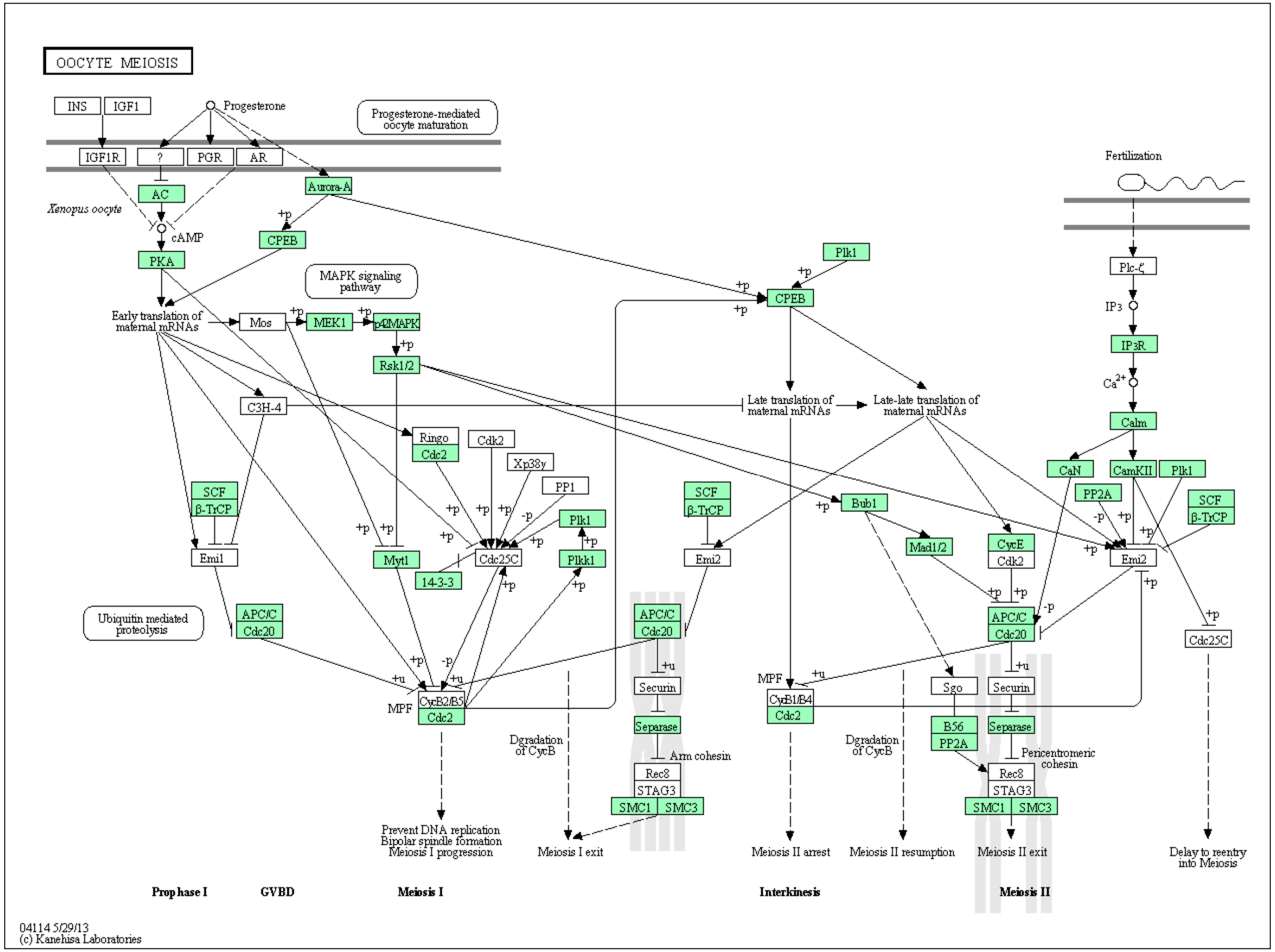

(**G**)

Figure S1. *Cont.*

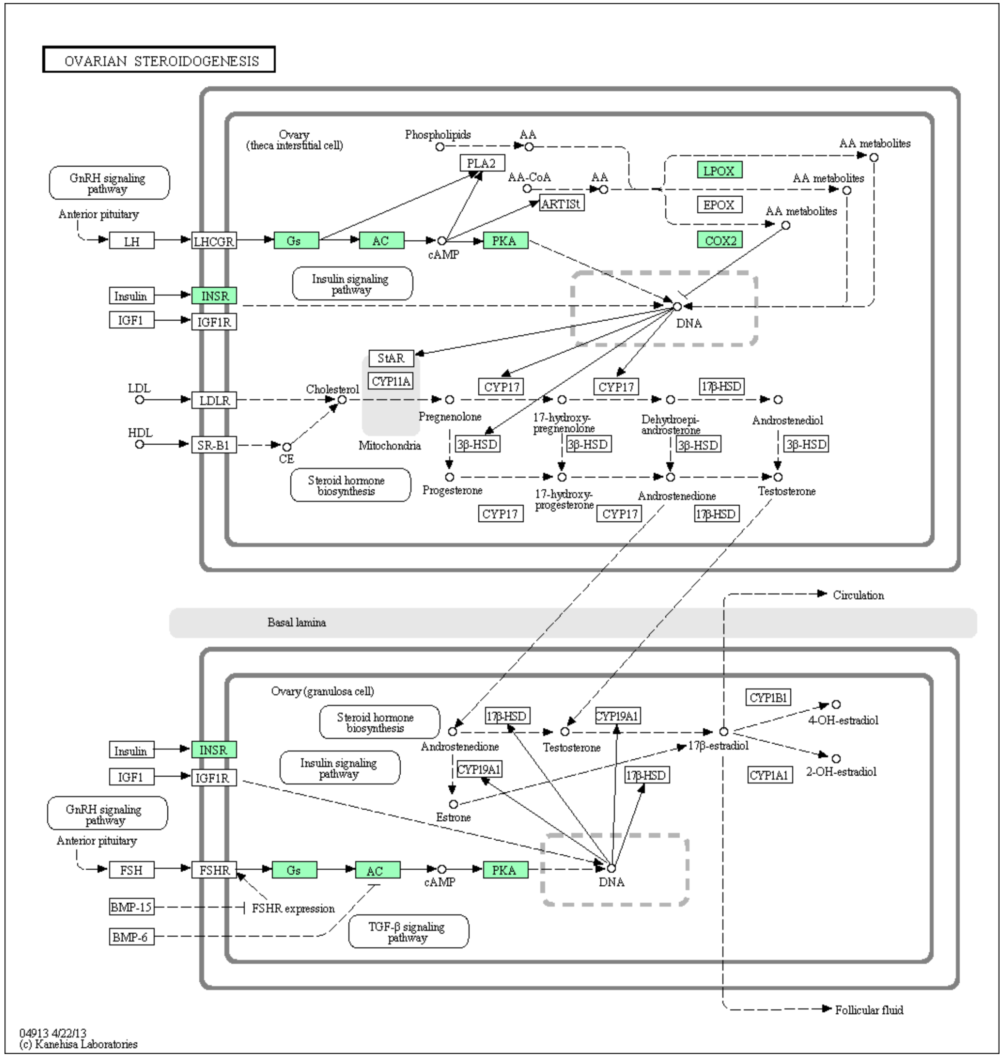

(**H**)

Figure S1. *Cont.*

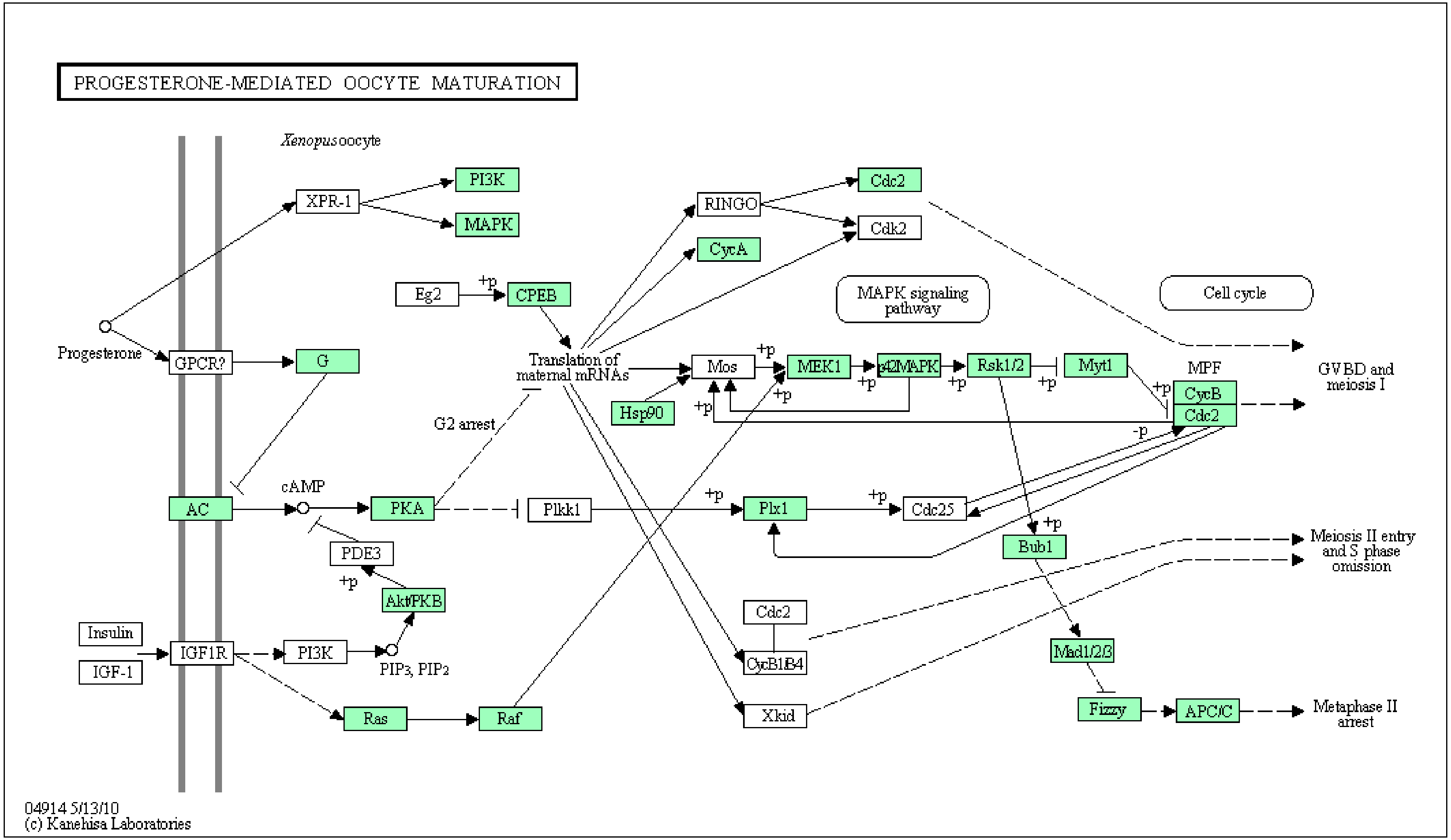

(I)

Figure S1. *Cont.*

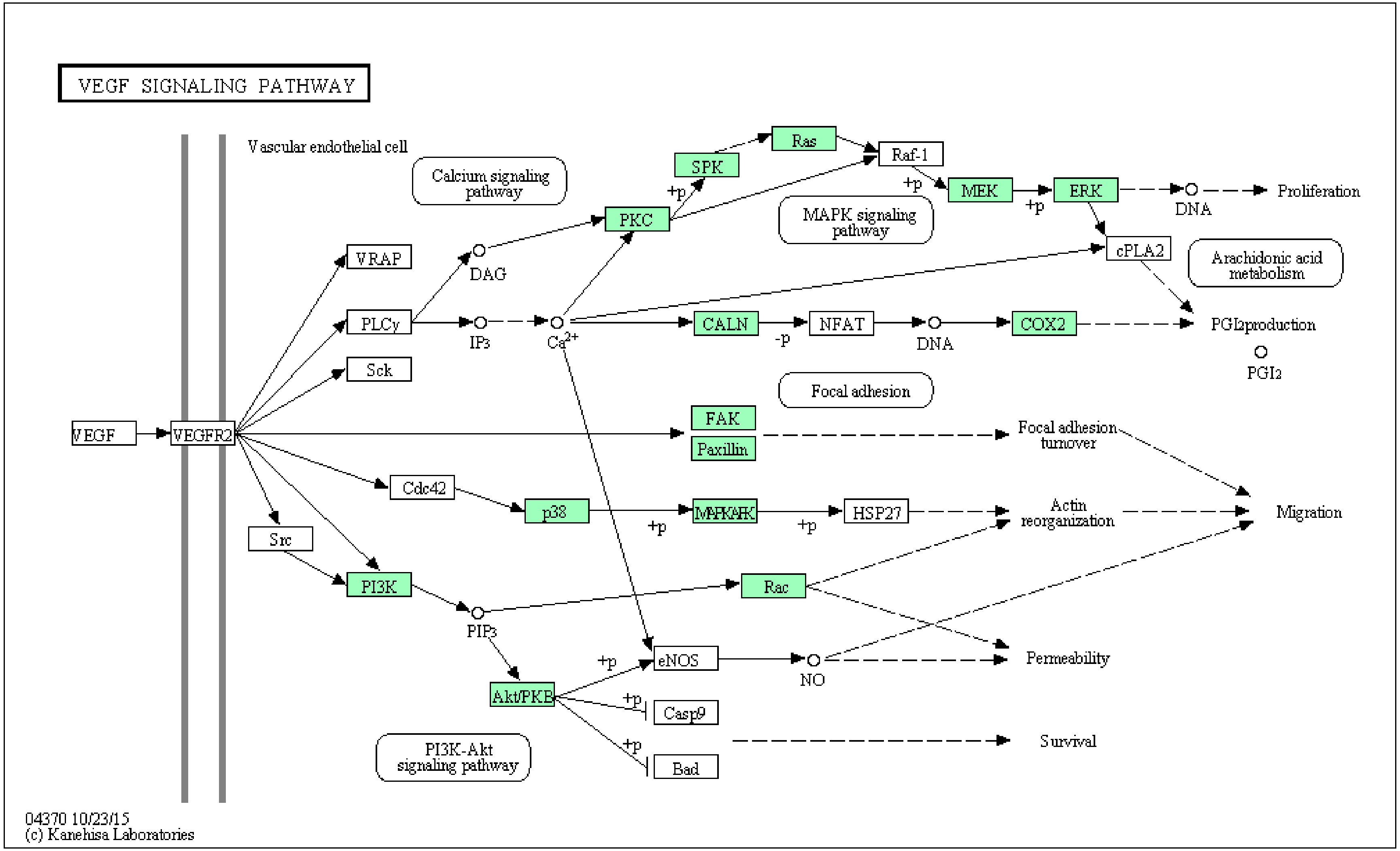

(J)

Figure S1. *Cont.*

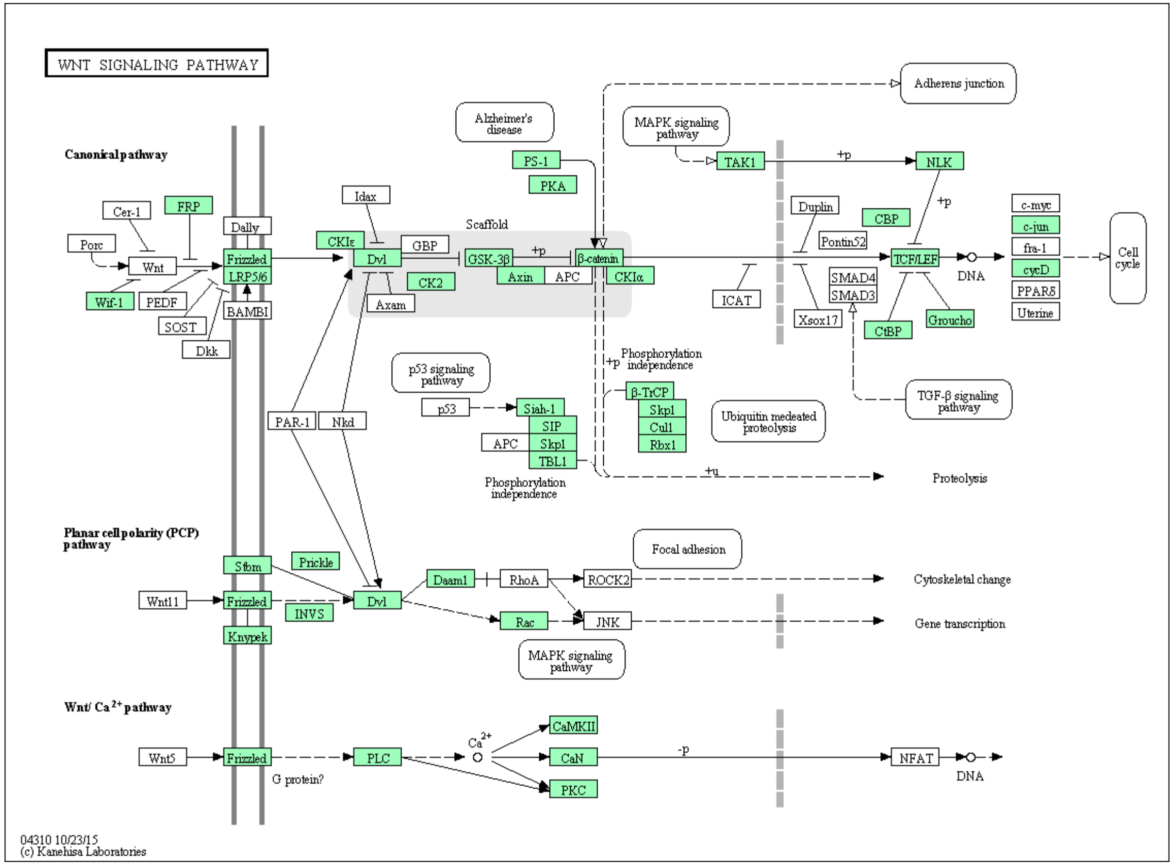

(**K**)

Figure S1. Summary of KEGG MAPs for all combined contigs in *M. rosenbergii*. Similarity of contigs generated with the CLC Genomic Workbench were searched and annotated using KAAS (KEGG Automatic Annotation Server). A few selected KEGG pathway maps potentially involved in sex differentiation and gonadal reproduction are presented. (**A**) ERBB signalling pathway; (**B**) estrogen signalling pathway; (**C**) GnRH signalling pathway; (**D**) insulin secretion; (**E**) insulin resistance; (**F**) insulin signalling pathway; (**G**) oocyte meiosis; (**H**) ovarian steroidogenesis; (**I**) progesterone-mediated oocyte maturation; (**J**) VEGF signalling pathway; (**K**) WNT signalling pathway.
